# Inflammatory Myofibroblastoma of the Breast: A Case Report

**DOI:** 10.3389/fonc.2021.646336

**Published:** 2021-06-01

**Authors:** Ling Wei, Guoyuan Jiang, Lala Bai, Tingchao Li, Xuejin Ma, Lin Jiang, Jie Wang, Shiguang Li

**Affiliations:** Department of Radiology, The Third Affiliated Hospital of Zunyi Medical University, The First People’s Hospital of Zunyi, Zunyi, China

**Keywords:** inflammatory myofibroblastoma, breast, mammography, MRI, case report

## Abstract

Inflammatory myofibroblastic tumor (IMT) is a rare tumor with low-grade malignant risk mainly occurring in soft tissues and lungs, and it is extremely rare in the breast. Meanwhile, imaging findings of the tumor often present with non-specific features that lead to misdiagnosis and delayed treatment. Here, we report a case of inflammatory myofibroblastic tumor in the breast with the imaging findings of mammography, magnetic resonance imaging (MRI), and pathologic findings to improve the understanding of the disease. The patient was treated by surgical operation, and was followed up for 44 months, no local recurrence and distant metastasis.

## Introduction

Inflammatory myofibroblastic tumor (IMT) of the breast, first reported at 1988 (1), is now considered as a true low-grade neoplasm and mixture of spindle cells and chronic inflammatory cells, such as lymphocytes, plasma cells, and eosinophils according to the 2013 World Health Organization classification of tumors of soft tissue (2). Although it is more often seen in the lung, soft tissue, and viscera in children and young adults, broad age range has been documented in recent years (3). The incidence of breast IMT is unknown, to our knowledge, approximately 30 cases have been described in the literature, one of which is reported to appear near a previous surgical site. The case we reported also occurred at the site of a previous operation. Breast IMTs are easily misdiagnosed and confused with other breast disorders like cancer or fibroadenoma due to lack of typical clinical and imaging characteristics. The purpose of this report is to shed new understanding on this rare tumor-like disorder by analyzing the complete pathological and imaging documentation of one breast IMT in our hospital.

## Case Report

A 50-year-old woman presented with a gradually increasing mass in the right breast, accompanied by progressive pain and slight redness of the localized skin. Two months ago, a quail egg-like mass was detected in the inner upper quadrant of the right breast when she underwent a physical examination, but she did not take any diagnostic and treatment measures. The woman had a history of surgery for fibroadenoma of the right breast two years ago, so it is worthy of being mentioned that the mass is just located right at the site of the previous surgery. Physical examination revealed a 5-cm old postoperative scar at the inner upper quadrant of the right breast, and a well-circumscribed mass measuring 4.5 cm in diameter was identified here with regular shape and slightly mobility. She had no family history of breast cancer. Ultrasonography (US) revealed an irregular shaped, hypoechoic mass with well-defined margins at the 2 o’clock position in the right breast, while septum and punctate blood flow signals were detected in the lesion. The lesion was classified as Breast Imaging Reporting and Data System (BI-RADS) category 4A.

**Figure 1:**
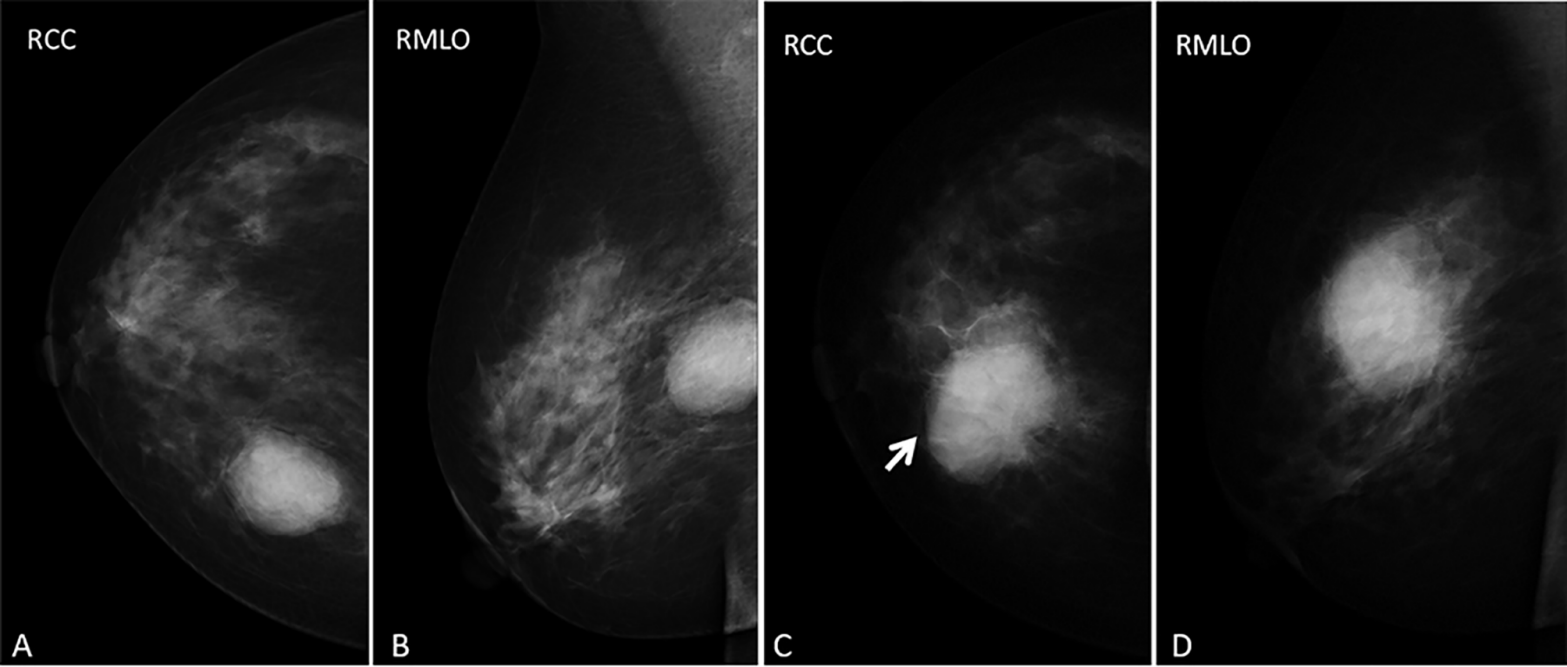
**(A–D)** images represent RCC and RMLO views before the first fibroadenoma surgery and in the current presentation (the inflammatory myofibroblastic tumor), respectively. Mammography obtained before fibroadenoma surgery showed **(A, B)** an oval-shaped, circumscribed mass and mammography performed two years after the operation showed **(C, D)** a slight hyperdense mass with an ill-defined, spiculated margin, accompanied by an incomplete halo sign (arrow) and thickened the skin adjacent to the areolar in the inner upper quadrant of the right breast.

Mammography obtained before fibroadenoma surgery showed a well-defined high-density mass in the inner upper quadrant of the right breast (**Figures 1A, B**). Mammography, performed two years after the initial operation, showed a high density, irregular mass with spiculated margins, accompanied by an incomplete halo sign (**Figures 1C** arrow, **D**) in the inner upper quadrant of the right breast. In addition, the skin adjacent to the areola had thickened. We classified the mass as BI-RADS category 4B according to imaging findings in mammography, including irregular shape, ill-defined margins, and incomplete halo sign around the mass.

**Figure 2:**
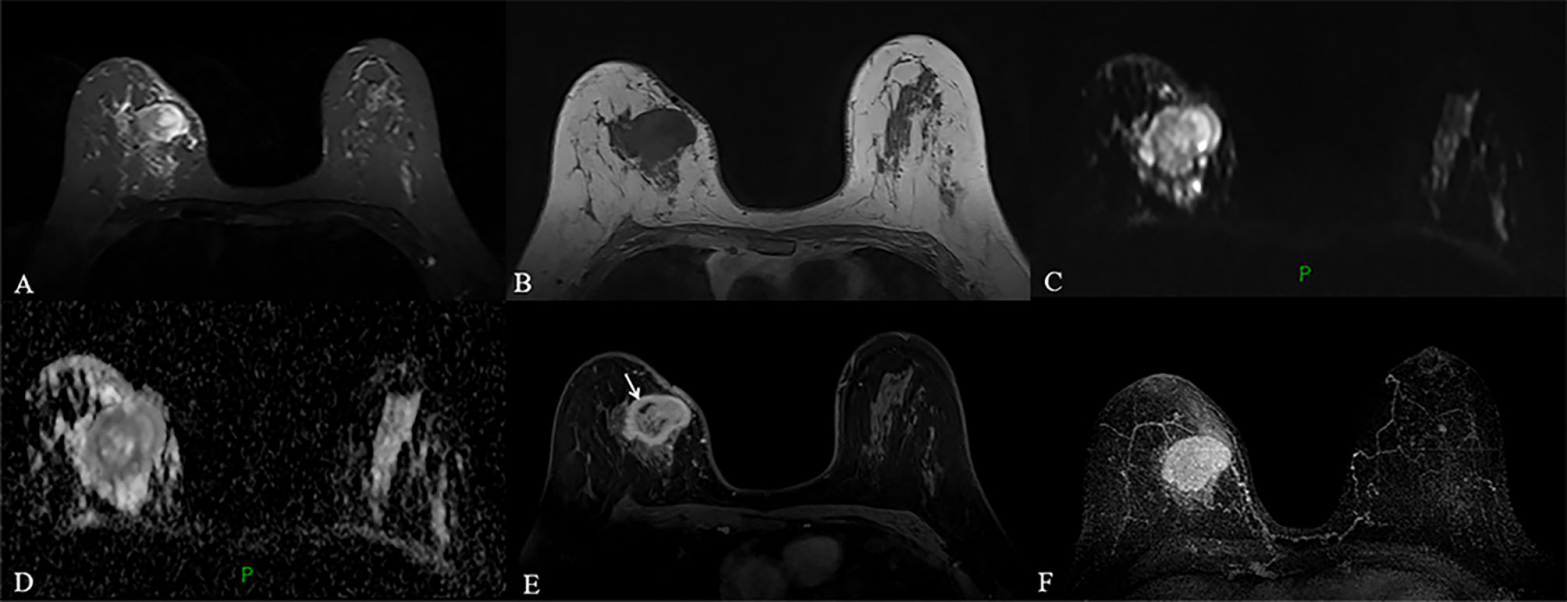
**(A–F)** MRI obtained two years after the operation revealed a circumscribed mass with irregular shape and slightly distorted structure, accompanied by thickening of adjacent skin in the inner upper quadrant of the right breast, a mass showed heterogeneous high signal intensity on fat-suppressed T2-weighted images and low signal intensity on T1-weighted images **(A, B)**. The lesion with high signal intensity on DWI **(C)** had low signal intensity on ADC map **(D)**, which indicated restricted diffusion. Dynamic contrast-enhanced MRI revealed a fast, heterogeneous enhanced mass in the initial phase **(E)**. Maximum intensity projection (MIP) revealed a mass with increased blood supply **(F)**.

**Figure 3:**
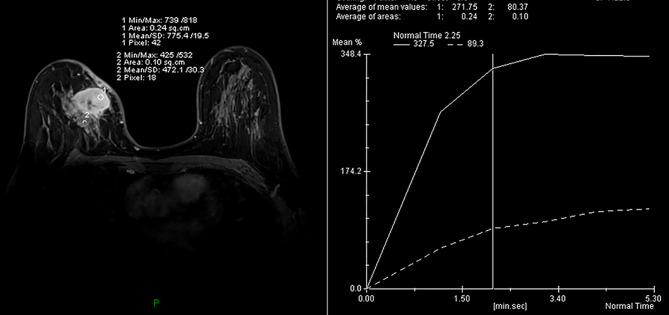
A time intensity curve of the mass showed a plateau (TIC II) in the delayed period.

MRI of the breast performed during a prior hospital admission, including traditional MRI and gadolinium-enhanced breast MRI, revealed an irregular mass, accompanied by architectural distortion, measuring 4.5 × 3.5 × 3.0 cm in size under the scar area of the inner upper quadrant of the right breast (**Figure 2**). The lesion had iso/hyper-intensity on axial fat-suppressed T2-weighted imaging (**Figure 2A**) and iso/hypo-intensity on axial T1-weighted imaging (**Figure 2B**), which showed heterogeneous high signal intensity on DWI (b = 1000 s/mm2) with low signal intensity ADC map (**Figures 2C, D**) that suggested restricted diffusion. Dynamic-contrast enhanced MRI showed a rapid heterogeneous rim enhancement mass with dark internal septation (**Figure 2E**, arrow), accompanied by non-mass enhancement in the initial period (**Figure 2E**), increased blood flow around the lesion in the maximal intensity projection (**Figure 2F**), and 201% of the early enhanced rate and a plateau in the delayed period and in the time intensity curve of the lesion (**Figure 3**). The lesion was assessed as BI-RADS category 4B by the readers, which suggested the likelihood of a malignant lesion. However, there was a history of surgery at the site of the mass two years ago and the clinical picture was characterized by progressive pain and mild skin redness. So a suspicion of inflammatory lesion was raised. After multidisciplinary discussion between breast surgeons and radiologists, the decision was made to proceed with surgical resection.

**Figure 4:**
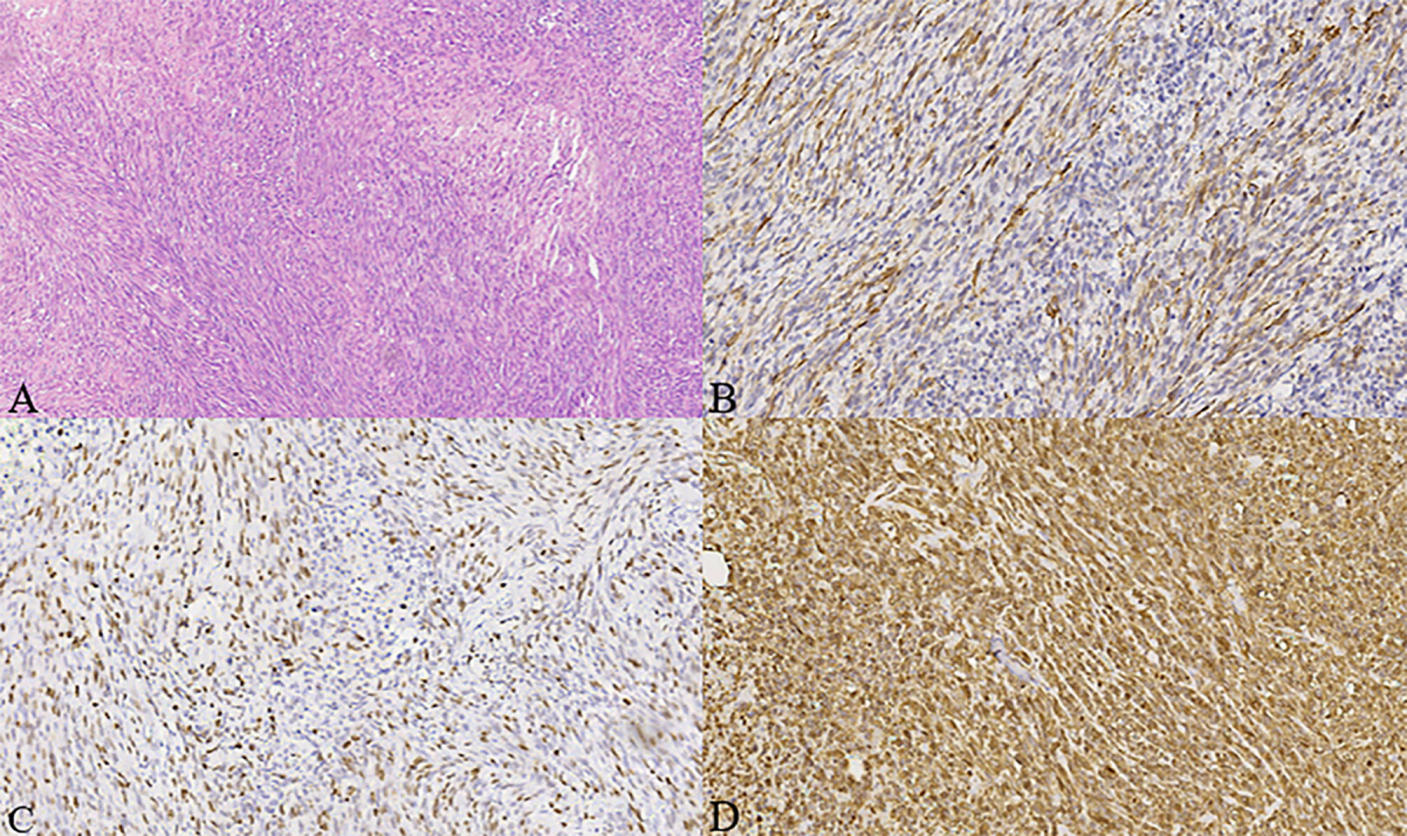
**(A–D)** Histologic, immunohistochemical staining of inflammatory myofibroblastic tumor (IMT). The mass showed spindle cells admixed with inflammatory cells (H&E stain, × 100) **(A)**. Immunohistochemical staining showed positive staining for smooth muscle actin (SMA) in the spindle tumor cells (SMA, × 200) **(B)**, positive staining for Ki-67 in about 30% of the spindle tumor cells (Ki-67, × 200) **(C)**, as well as positive staining for vimentin in the spindle tumor cells (vimentin, × 200) **(D)**.

**Figure 5:**
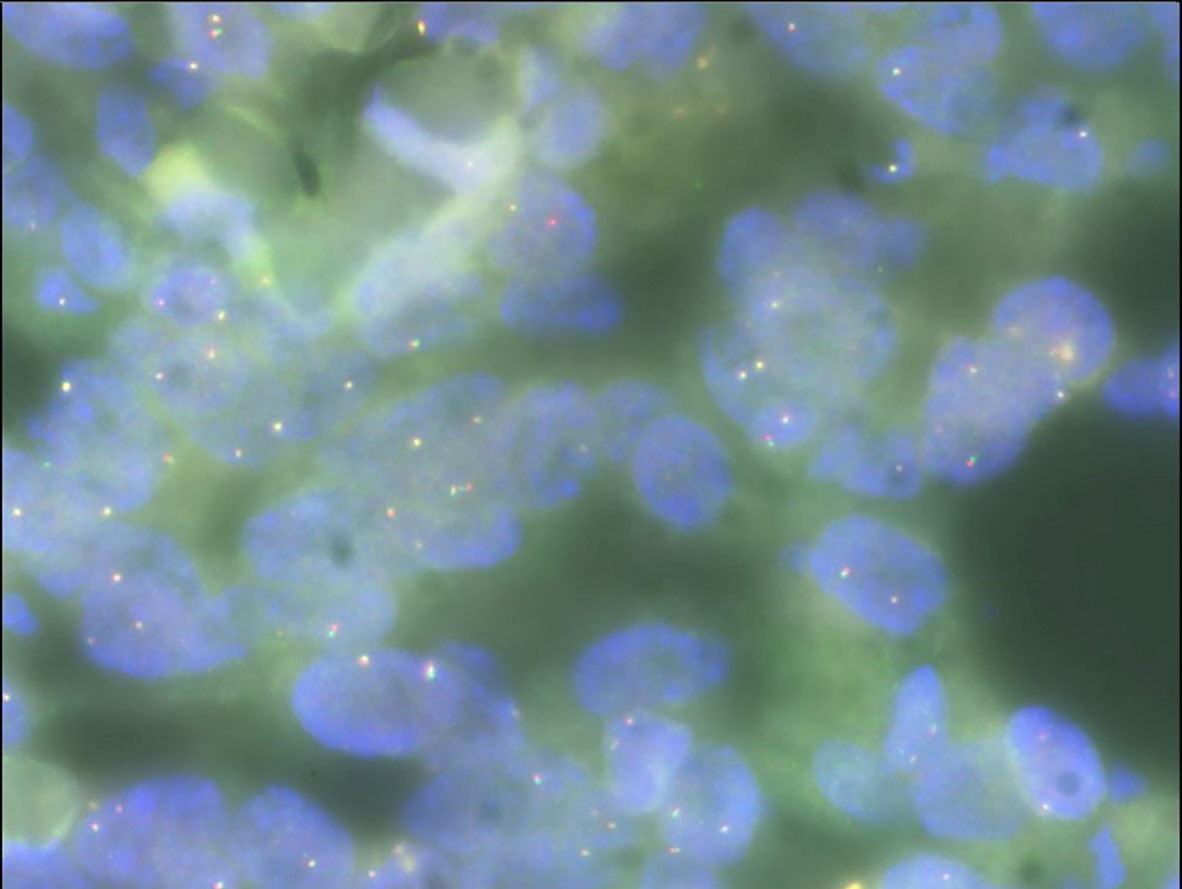
Fluorescence *in situ* hybridization analysis showing no ALK gene rearrangement (× 100).

Gross examination of the surgical specimen revealed a 5 × 4 × 4 cm mass that was solid and greyish-white and yellowish. Pathologic analysis of the mass showed proliferating spindle cells interwoven within inflammatory cells, including plasma cells and lymphocytes (**Figure 4A**). On immunohistochemical examination (**Figure 4**), the mass was positive for smooth muscle actin (SMA) (**Figure 4B**), vimentin (**Figure 4D**), and Ki-67 in about 30% of the spindle tumor cells (**Figure 4C**) while negative for CD34. Meanwhile, no ALK gene rearrangement was detected by fluorescence *in situ* hybridization (**Figure 5**). The diagnosis of inflammatory myofibroblastic tumor (IMT) was established by the pathological findings.

## Discussion

Inflammatory myofibroblastic tumor of the breast is an extremely rare entity that can recur, with a recurrence rate of 25% (4). So far, the pathogenesis of IMT is still unclear, and it was initially thought to be related to trauma, infection, surgery, and other stimuli, which finally activated myofibroblasts with proliferative potential to proliferate and form tumors, and were called inflammatory pseudotumors. However, recent studies have shown that both intrapulmonary and extrapulmonary IMT are abnormal with chromosomes 2 and 9. Approximately 50% ~ 75% of IMT showed the fusion of ALK, TPM3, and TPM4 genes on 2P23, leading to the overexpression of the ALK protein (5, 6). Some groups reported that IMT was related to the expression of p53 and MDM2 genes (7). ALK gene fusion caused by a non-classical pathway was also found in IMT, suggesting that the ALK signaling pathway plays an important role in the majority of IMT tumors (8, 9). These findings suggest that IMT is a true tumor and not an inflammatory lesion, and that genetics and molecular studies confirm that IMT is a monoclonal proliferation.

At present, the diagnosis of the disease is challenging. In this regard, we make some differential diagnoses of some breast diseases according to the imaging and pathology of the case to improve the understanding of the disease. Based on this case, many other causes of breast mass may be considered in differential diagnoses.

Fibroadenoma and phyllodes tumor should firstly be included in the differential diagnosis due to the black septum and lobulated shape of the mass shown on contrast-enhanced MRI. Duman L et al. (10) reported that fibroadenoma tended to be homogeneously enhanced and had a hypointense internal septum, while in this case, even with a hypointense septum, the mass showed rapid heterogeneous enhancement which made a fibroadenoma unlikely. Although phyllode tumors are frequently lobulated in shape–this is similar to the MRI findings in this case–the internal cystic areas were detected in the former. Imaging findings of the two diseases are non-specific, hence final diagnoses are based on histopathological findings. A phyllode tumor is a tumor in which epithelial cells and stromal cells grow simultaneously. It is diagnosed when the fibroepithelial architecture shows an inflated intracanalicular pattern with leaf-like fronds protruding into cystically dilated spaces accompanied by hypercellularity (11). So, a phyllode tumor was excluded from the differential diagnosis.

Meanwhile, the differential diagnosis may secondly include spindle cell lesions, either benign or malignant. Myofibroblastomas of the breast (MFB) are common in men, and their clinical manifestations are a steady and slow-growing mass with the duration of months to years. Imaging manifestations of MFB are usually benign, showing high signal and internal septum on T2WI without restricted diffusion, but malignancy cannot be ruled out safely just with MRI. The diagnosis is mainly based on immunohistochemical analysis including positive results for SMA, CD34, CD10, and desmin whereas CD117 is usually negative (12, 13).

Nodular fasciitis (NF) of the breast is a benign, pseudomyofibroblastic proliferative lesion. Rapid growth and spontaneous dissipation may be the characteristics of NF. The typical imaging findings of NF include low or iso-signal intensity on T1WI and hyperintensity on T2WI with enhancement after contrast injection (14). These features are not specific, however, its diagnosis often depends on pathology. It is reported in the literature that SMA and CD38 are positive, vimentin is positive, and AE1/AE3 are negative (15).

Breast fibromatosis is a locally invasive benign tumor with a high recurrence tendency and no metastasis potential, which can occur in the breast parenchyma, or originate from the pectoral fascia and extend into the breast. The typical manifestation of breast fibromatosis is a mass with unclear boundary and irregular shape, which sometimes may be accompanied by stretching and binding of the Cooper ligament. Fascia involvement may be the feature of breast fibromatosis (16). Although the imaging findings of breast fibromatosis are unspecific, MRI can be used to evaluate the location and degree of lesion before operation, and to evaluate residual lesions and detect recurrence after operation. The typical immunohistochemical features of fibromatosis are localized expression of SMA and calcium protein, but no cytokeratin CD34 or S100. Almost all reported cases had focal expression of beta catenin (17).

Spindle cell carcinoma to the breast (SpCC) usually manifests as a rapidly growing mass, however, their prominent clinical manifestations remain unclear. A high density ill-defined mass is seen on mammography whereas an irregular mass with microlobulated margin, complex echogenicity, and posterior acoustic enhancement on ultrasound imaging are common features in the diagnostic images of SpCC (18). The evidence of diffuse cytokeratin or p63 immunoreactivity in the malignant spindle cells suggests a diagnosis of spindle cell breast carcinoma (19, 20).

We present a rare case of inflammatory myofibroblastoma that may be associated with trauma of the breast, similar cases have been reported where the lesion occurred near the postoperative site of fibroadenoma in the literature (21), which may provide some instruction for future clinical work: if a patient with a history of surgery has lesions in the surgical site, inflammatory myofibroblastic tumor may be included in the diagnosis. The patient was followed for 44 months without recurrence or metastasis.

In conclusion, IMT of the breast is a very rare condition. Its clinical findings are mostly related to inflammatory conditions but some of the features are more similar to the lines of the tumor pathology. Even if it is a low grade malignant tumor, recurrence and metastasis may be picked up by systematic follow-up and for this reason it is treated usually by surgical resection. Therefore, it is so important to determine the correct diagnosis and treatment of this condition.

## Data Availability Statement

The original contributions presented in the study are included in the article/supplementary material. Further inquiries can be directed to the corresponding author.

## Ethics Statement

Written informed consent was obtained from the individual(s) for the publication of any potentially identifiable images or data included in this article.

## Author Contributions

LW and GJ: manuscript writing. TL: pathological review. LB, LJ, XM, and JW: manuscript revision. SL: conception and critical review. All authors contributed to the article and approved the submitted version.

## Conflict of Interest

The authors declare that the research was conducted in the absence of any commercial or financial relationships that could be construed as a potential conflict of interest.
